# Estimating Distributions of Parameters in Nonlinear State Space Models with Replica Exchange Particle Marginal Metropolis–Hastings Method

**DOI:** 10.3390/e24010115

**Published:** 2022-01-12

**Authors:** Hiroaki Inoue, Koji Hukushima, Toshiaki Omori

**Affiliations:** 1 Graduate School of Engineering, Kobe University, 1-1 Rokkodai-cho, Nada-ku, Kobe 657-8501, Japan; 169t804t@stu.kobe-u.ac.jp; 2 Graduate School of Arts and Sciences, The University of Tokyo, 3-8-1 Komaba, Meguro-ku, Tokyo 153-8902, Japan; k-hukushima@g.ecc.u-tokyo.ac.jp; 3 Komaba Institute for Science, The University of Tokyo, 3-8-1 Komaba, Meguro-ku, Tokyo 153-8902, Japan; 4 Organization for Advanced and Integrated Research, Kobe University, 1-1 Rokkodai-cho, Nada-ku, Kobe 657-8501, Japan; 5 Center for Mathematical and Data Sciences, Kobe University, 1-1 Rokkodai-cho, Nada-ku, Kobe 657-8501, Japan

**Keywords:** state space model, probabilistic graphical model, replica exchange particle Metropolis–Hastings method, replica exchange method, particle Metropolis–Hastings method, particle Markov chain Monte Carlo method

## Abstract

Extracting latent nonlinear dynamics from observed time-series data is important for understanding a dynamic system against the background of the observed data. A state space model is a probabilistic graphical model for time-series data, which describes the probabilistic dependence between latent variables at subsequent times and between latent variables and observations. Since, in many situations, the values of the parameters in the state space model are unknown, estimating the parameters from observations is an important task. The particle marginal Metropolis–Hastings (PMMH) method is a method for estimating the marginal posterior distribution of parameters obtained by marginalization over the distribution of latent variables in the state space model. Although, in principle, we can estimate the marginal posterior distribution of parameters by iterating this method infinitely, the estimated result depends on the initial values for a finite number of times in practice. In this paper, we propose a replica exchange particle marginal Metropolis–Hastings (REPMMH) method as a method to improve this problem by combining the PMMH method with the replica exchange method. By using the proposed method, we simultaneously realize a global search at a high temperature and a local fine search at a low temperature. We evaluate the proposed method using simulated data obtained from the Izhikevich neuron model and Lévy-driven stochastic volatility model, and we show that the proposed REPMMH method improves the problem of the initial value dependence in the PMMH method, and realizes efficient sampling of parameters in the state space models compared with existing methods.

## 1. Introduction

Extracting latent nonlinear dynamics from observed time-series data is important for understanding the dynamic system against the background of the observed data. A state space model is a probabilistic graphical model for time-series data that assumes the existence of latent variables that cannot be observed directly [1,2,3,4,5,6,7,8,9,10,11,12,13,14,15,16,17,18,19,20,21,22,23,24,25]. State space models are used in various fields to forecast observation values [7,15,22] and to estimate latent variables [11,20,21,26]. In many cases, however, model parameters are unknown. Therefore, estimating the model parameters from observations is an important task for the state space models.

To estimate the parameters in the state space models from observations, a method combining the sequential Monte Carlo (SMC) method [2,4,8,9,10,11,12,14,16,17,18,19,20,23,24,25,27] with the expectation–maximization (EM) algorithm [8,28,29] has been proposed [9,10,11,14,20]. This method is based on a maximum likelihood estimation framework, and it estimates parameters by sequentially updating the parameters so that the likelihood increases. Although it is guaranteed that a local optimum can be estimated by iteratively updating the parameters, the global optimum may not be estimated depending on the initial values of parameters. Furthermore, since the EM algorithm is a point estimation method, it is not possible to identify whether converged values are local or global optima.

To estimate the distribution of parameters in a state space model, two kinds of particle Markov chain Monte Carlo (PMCMC) methods have been proposed: the particle Gibbs (PG) method and the particle marginal Metropolis–Hastings (PMMH) method [12]. Both methods combine Markov chain Monte Carlo (MCMC) methods with the SMC method, and the distribution of parameters is estimated by collecting samples. The PG method combines the SMC method with Gibbs sampling [8,30], and the PG method samples latent variables and parameters in a state space model from the joint posterior distribution of latent variables and parameters alternately. In the PG method, the SMC method is employed for sampling latent variables. The PMMH method, on the other hand, combines the SMC method with the Metropolis–Hastings (MH) algorithm [8,25,31,32], and the PMMH method samples parameters in a state space model from the marginal posterior distribution of parameters. In the PMMH method, the SMC method is employed for calculating the likelihood marginalized over the distribution of latent variables. Both the PG method and the PMMH method have been widely applied (for example, the PG method [33,34], the PMMH method [35,36,37]).

In recent years, some extended versions of PG methods have been proposed to improve the sampling efficiency and initial value dependence, such as the particle Gibbs with ancestor sampling (PGAS) method and the replica exchange particle Gibbs with ancestor sampling (REPGAS) method [16,18,19,24]. Thus far, however, the existing methods for such improvement have been proposed for only the PG method. Therefore, it is important to improve the PMMH method for the accurate estimation of parameters since the PMMH method may have the problem of the initial value dependence.

In this paper, we propose the replica exchange particle marginal Metropolis–Hastings (REPMMH) method, which combines the PMMH method with the replica exchange method [24,38,39,40] in order to improve the problem of initial value dependence in the PMMH method. Combining the replica exchange method with the PMMH method makes it possible to estimate the parameters governing the dynamics for very complex and nonlinear time-series data. We first describe the state space models and explain the PMMH method as a conventional method. Then, after explaining our proposed method, we conduct experiments to compare the proposed method with the conventional methods, the PMMH method and the REPGAS method.

## 2. Methods

In this section, we propose our replica exchange particle marginal Metropolis–Hastings (REPMMH) method. First, we describe a state space model for time-series data using a probabilistic graphical model. Next, we describe the conventional particle marginal Metropolis–Hastings (PMMH) method to estimate the marginal posterior distribution of parameters obtained by marginalization over the distribution of latent variables in a state space model. After this, we propose the REPMMH method, which combines the PMMH method with the replica exchange method to improve the problem of initial value dependence in the PMMH method.

### 2.1. State Space Model

**Figure 1:**
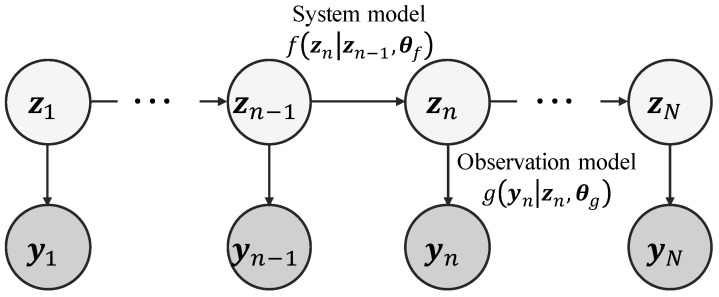
Probabilistic graphical model of a state space model. $z_{1:N}=\left\{z_{1},z_{2},\ldots ,z_{N}\right\}$ and $y_{1:N}=\left\{y_{1},y_{2},\ldots ,y_{N}\right\}$, respectively, represent latent variables and observations for time step n=1,2,…,N. The arrow to the latent variables $z_{n}$ at the time step *n* from the latent variables $z_{n-1}$ at the previous time step n−1 represents a system model $f\left(z_{n}|z_{n-1},\theta_{f}\right)$, and the arrow to the observations $y_{n}$ at the time step *n* from the latent variables $z_{n}$ at the time step *n* represents an observation model $g\left(y_{n}|z_{n},\theta_{g}\right)$. $\Theta =\left\{\theta_{f},\theta_{g}\right\}$ are parameters to be estimated.

We show a state space model as a probabilistic graphical model in Figure 1. Note that there are two type of variables, latent variables $z_{1:N}=\left\{z_{1},z_{2},\ldots ,z_{N}\right\}$ and observations $y_{1:N}=\left\{y_{1},y_{2},\ldots ,y_{N}\right\}$, at the time steps $\left\{1,2,\ldots ,N\right\}$ in the state space model. The latent variables $z_{1:N}$ cannot be observed directly and only the observations $y_{1:N}$ are observable. At a time step *n*, the state space model is represented as follows:

(1) $$\begin{array}{c} z_{n}∼f\left(z_{n}|z_{n-1},\theta_{f}\right), \end{array}$$

(2) $$\begin{array}{c} y_{n}∼g\left(y_{n}|z_{n},\theta_{g}\right), \end{array}$$

where $f\left(z_{n}|z_{n-1},\theta_{f}\right)$ and $g\left(y_{n}|z_{n},\theta_{g}\right)$ are called a system model and an observation model, respectively. $\theta_{f}$ are the parameters of the system model, and $\theta_{g}$ are the parameters of the observation model. The system model $f\left(z_{n}|z_{n-1},\theta_{f}\right)$ represents the process of updating the latent variables $z_{n}$ from the previous latent variables $z_{n-1}$. Moreover, the observation model $g\left(y_{n}|z_{n},\theta_{g}\right)$ represents the process of obtaining observations $y_{n}$ from the latent variables $z_{n}$.

The goal of this paper is to estimate the posterior distribution of the parameters $p\left(\Theta |y_{1:N}\right)$ for the given observations $y_{1:N}$, where Θ is represented as $\Theta =\left\{\theta_{f},\theta_{g}\right\}$. However, since the latent variables exist in the state space models, it is necessary to perform marginalization with respect to the latent variables in order to obtain the marginal posterior distribution $p\left(\Theta |y_{1:N}\right)$. Because it is often difficult to calculate the marginal posterior distribution $p\left(\Theta |y_{1:N}\right)$ analytically, we propose a new method for estimating the marginal posterior distribution $p\left(\Theta |y_{1:N}\right)$ based on the PMMH method in this paper.

### 2.2. Particle Marginal Metropolis–Hastings Method

The PMMH method combines the sequential Monte Carlo (SMC) method [2,4,8,9,10,11,12,14,16,17,18,19,20,23,24,25,27] with the Metropolis–Hastings (MH) algorithm [8,25,31,32]. The PMMH method was proposed to estimate the marginal posterior distribution of parameters $p\left(\Theta |y_{1:N}\right)$ for time-series observations $y_{1:N}$ represented as a state space model [12].

In the PMMH method, the marginal likelihood $p\left(y_{1:N}|\Theta \right)$ is used to evaluate the appropriateness of parameters Θ. Here, the SMC method is used to calculate the marginal likelihood $p\left(y_{1:N}|\Theta \right)$ of the parameters Θ obtained by marginalization over the distribution of latent variables $z_{1:N}$. A new sample candidate of parameters $\Theta^{*}=\left\{\theta_{f}^{*},\theta_{g}^{*}\right\}$ is proposed from an arbitrary proposal distribution $q\left(\Theta |\Theta \left[k-1\right]\right)$ given the sample one step before $\Theta \left[k-1\right]$, where *k* is the sample number. Moreover, whether to accept or reject the sample candidate $\Theta^{*}$ is determined based on the following acceptance probability:

(3) $$p_{\mathrm{accept}}=\min\left(1,\frac{p\left(y_{1:N}|\Theta^{*}\right)p\left(\Theta^{*}\right)}{p\left(y_{1:N}|\Theta \left[k-1\right]\right)p\left(\Theta \left[k-1\right]\right)}\frac{q\left(\Theta \left[k-1\right]|\Theta^{*}\right)}{q\left(\Theta^{*}|\Theta \left[k-1\right]\right)}\right),$$

where $p\left(\Theta \right)$ represents the prior distribution of parameters. $p\left(y_{1:N}|\Theta \right)$ is the marginal likelihood obtained by marginalization over the distributions of latent variables $z_{1:N}$ as follows:

(4) $$\begin{array}{ccc} p\left(y_{1:N}|\Theta \right) & = & \int p\left(y_{1:N},z_{1:N}|\Theta \right)dz_{1:N}\\ & = & \int g\left(y_{1}|z_{1},\theta_{g}\right)p\left(z_{1}\right)\prod_{n=2}^{N}g\left(y_{n}|z_{n},\theta_{g}\right)f\left(z_{n}|z_{n-1},\theta_{f}\right)dz_{1:N}, \end{array}$$

where $p\left(z_{1}\right)$ is the distribution of latent variables $z_{1}$ at time step 1. Since it is difficult to obtain the marginal likelihood $p\left(y_{1:N}|\Theta \right)$ analytically, the SMC method is used in the PMMH method to calculate the marginal likelihood $p\left(y_{1:N}|\Theta \right)$ numerically.

The SMC method estimates the distribution of latent variables by approximating the distribution with the density of the particles $\left\{z_{1:N}^{\left(1\right)},z_{1:N}^{\left(2\right)},\ldots ,z_{1:N}^{\left(M\right)}\right\}$ as follows:

(5) $$p\left(z_{1:N}|y_{1:N},\Theta \right)≃\frac{1}{M}\sum_{m=1}^{M}\delta \left(z_{1:N}-z_{1:N}^{\left(m\right)}\right),$$

where $z_{1:N}^{\left(m\right)}$ is the *m*-th particle and *M* is the number of particles. $\delta \left(z_{1:N}\right)$ is the Dirac delta distribution.

To obtain particles $\left\{z_{n}^{\left(1\right)},z_{n}^{\left(2\right)},\ldots ,z_{n}^{\left(M\right)}\right\}$ at a time step *n*, we sample the *m*-th particle $z_{n}^{\left(m\right)}$ at the time step *n* from the *m*-th particle $z_{n-1}^{\left(m\right)}$ at the previous time step n−1 for each $m\in \left\{1,2,\ldots ,M\right\}$ with the system model as follows:

(6) $$z_{n}^{\left(m\right)}∼f\left(z_{n}|z_{n-1}^{\left(m\right)},\theta_{f}\right).$$

Moreover, the obtained particles $\left\{z_{n}^{\left(1\right)},z_{n}^{\left(2\right)},\ldots ,z_{n}^{\left(M\right)}\right\}$ are resampled based on the normalized weights $\left\{W_{n}^{\left(1\right)},W_{n}^{\left(2\right)},\ldots ,W_{n}^{\left(M\right)}\right\}$ obtained as follows:

(7) $$W_{n}^{\left(m\right)}=\frac{w_{n}^{\left(m\right)}}{\sum_{l=1}^{M}w_{n}^{\left(l\right)}},$$

(8) $$w_{n}^{\left(m\right)}=g\left(y_{n}|z_{n}^{\left(m\right)},\theta_{g}\right).$$

By iterating the above flow for time step $n\in \left\{1,2,\ldots ,N\right\}$, particles that approximate the distribution of latent variables $z_{1:N}$ can be obtained. Here, the marginal likelihood $p\left(y_{1:N}|\Theta \right)$ can be calculated approximately as follows:

(9) $$p\left(y_{1:N}|\Theta \right)=\prod_{n=1}^{N}p\left(y_{n}|y_{1:n-1},\Theta \right)≃\frac{1}{M}\prod_{n=1}^{N}\sum_{m=1}^{M}w_{n}^{\left(m\right)}.$$

By calculating the acceptance probability $p_{\mathrm{accept}}$ in Equation (3) with the marginal likelihood $p\left(y_{1:N}|\Theta^{*}\right)$ for the sample candidate $\Theta^{*}$ obtained by Equation (9), it is determined whether to accept or reject the proposed sample candidate $\Theta^{*}$. We show the flow of the PMMH method described above in Algorithm 1.
**Algorithm 1** Particle Marginal Metropolis–Hastings (PMMH) Method.1:initialize the parameters $\Theta \left[0\right]$2:**for**k=1,…,K (*K* is the number of samples) **do**3:   draw the sample candidate of parameters $\Theta^{*}∼q\left(\Theta^{*}|\Theta \left[k-1\right]\right)$4:   draw the initial particles $z_{1}^{\left(m\right)}∼p\left(z_{1}\right)$ for m=1,…,M (*m* is the particle number of the particle that is the source of resampling)5:   calculate the weights of particles $\left\{w_{1}^{\left(1\right)},w_{1}^{\left(2\right)},\ldots ,w_{1}^{\left(M\right)}\right\}$ with Equation (8)6:   normalize the weights of particles $\left\{W_{1}^{\left(1\right)},W_{1}^{\left(2\right)},\ldots ,W_{1}^{\left(M\right)}\right\}$ with Equation (7)7:   resample the particles $\left\{z_{1}^{\left(1\right)},z_{1}^{\left(2\right)},\ldots ,z_{1}^{\left(M\right)}\right\}$ according to the normalized weights $\left\{W_{1}^{\left(1\right)},W_{1}^{\left(2\right)},\ldots ,W_{1}^{\left(M\right)}\right\}$8:   **for** n=2,…,N **do**9:      draw the particles $\left\{z_{n}^{\left(1\right)},z_{n}^{\left(2\right)},\ldots ,z_{n}^{\left(M\right)}\right\}$ at time step *n* with Equation (6)10:     calculate the weights of particles $\left\{w_{n}^{\left(1\right)},w_{n}^{\left(2\right)},\ldots ,w_{n}^{\left(M\right)}\right\}$ with Equation (8)11:     normalize the weights of particles $\left\{W_{n}^{\left(1\right)},W_{n}^{\left(2\right)},\ldots ,W_{n}^{\left(M\right)}\right\}$ with Equation (7)12:     resample the particles $\left\{z_{n}^{\left(1\right)},z_{n}^{\left(2\right)},\ldots ,z_{n}^{\left(M\right)}\right\}$ according to the normalized weights $\left\{W_{n}^{\left(1\right)},W_{n}^{\left(2\right)},\ldots ,W_{n}^{\left(M\right)}\right\}$13:   **end for**14:   calculate the marginal likelihood $p\left(y_{1:N}|\Theta^{*}\right)$ with Equation (9)15:   calculate the acceptance probability $p_{\mathrm{accept}}$ with Equation (3)16:   draw a uniform random number $\alpha ∼U\left(0,1\right)$ ($U\left(a,b\right)$ is a uniform distribution with range [a,b))17:   **if** $\alpha \leq p_{\mathrm{accept}}$ **then**18:     set the sample of parameters $\Theta \left[k\right]\leftarrow \Theta^{*}$19:   **else**20:     set the sample of parameters $\Theta \left[k\right]\leftarrow \Theta \left[k-1\right]$21:   **end if**22:**end for**23:**return**${\left\{\Theta \left[k\right]\right\}}_{k=1}^{K}$

### 2.3. Proposed Method

In our study, we propose the REPMMH method, which combines the PMMH method with the replica exchange method [24,38,39,40] to improve the problem of initial value dependence in the PMMH method. By employing the REPMMH method, we estimate the marginal posterior distribution of parameters from the time-series observations.

#### 2.3.1. Brief Summary of Our Proposed Method

**Figure 2:**
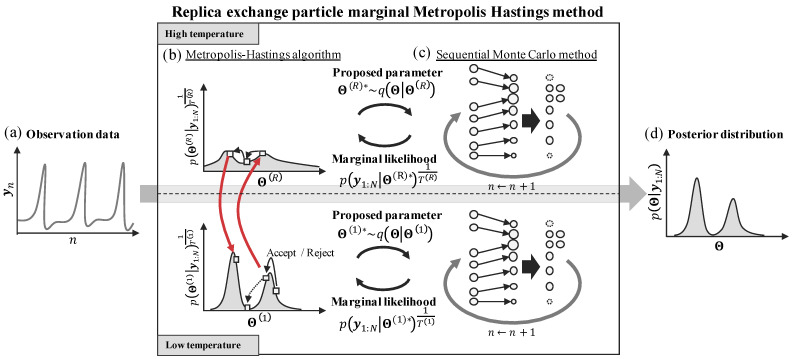
Schematic diagrams of the proposed replica exchange particle marginal Metropolis–Hastings (REPMMH) method. (**a**) The time-series observations $y_{1:N}$ as inputs. (**b**,**c**) The REPMMH method consisting of (**b**) the Metropolis–Hastings (MH) algorithms and (**c**) the sequential Monte Carlo (SMC) methods parallelly conducted at multiple temperatures. In the SMC method, the sample candidate $\Theta^{\left(r\right)*}$ proposed by the MH algorithm is used to obtain the marginal likelihood $p{\left(y_{1:N}|\Theta^{\left(r\right)*}\right)}^{\frac{1}{T^{\left(r\right)}}}$. By the SMC method, the marginalization over time-series of latent variables $z_{1:N}$ is conducted iteratively for time steps n=1,2,…,N. In the MH algorithm, the marginal likelihood $p{\left(y_{1:N}|\Theta^{\left(r\right)*}\right)}^{\frac{1}{T^{\left(r\right)}}}$ is used to determine whether to accept or reject the sample candidate. In the REPMMH method, exchanges between samples at different temperatures are considered in order to achieve the transitions that are difficult to achieve with the particle marginal Metropolis–Hastings (PMMH) method. The transitions can be realized by passing through a high temperature due to exchange between temperatures, as shown by the red arrows in the MH algorithm. (**d**) The estimated posterior distributions of parameters Θ as the output.

We show the schematic diagram of the REPMMH method in Figure 2. In our proposed REPMMH method, we introduce multiple different replicas of parameters $\left\{\Theta \right\}=\left\{\Theta^{\left(1\right)},\Theta^{\left(2\right)},\ldots ,\Theta^{\left(r\right)},\ldots ,\Theta^{\left(R\right)}\right\}$ at temperatures $T=\left[T^{\left(1\right)},T^{\left(2\right)},\ldots ,T^{\left(r\right)},\ldots ,T^{\left(R\right)}\right]$ into the PMMH method. As shown in the middle part of Figure 2, we employ the PMMH method in parallel at each temperature. In the PMMH method at each temperature $T^{\left(r\right)}$, we obtain the respective marginal likelihood $p{\left(y_{1:N}|\Theta^{\left(r\right)*}\right)}^{\frac{1}{T^{\left(r\right)}}}$ by employing the SMC method (Figure 2c) with the respective sample candidate $\Theta^{\left(r\right)*}$ proposed in the MH algorithm (Figure 2b).

For each temperature $T^{\left(r\right)}$, the SMC method and the MH algorithm are conducted as follows. In the SMC method (Figure 2c), the marginal likelihood $p{\left(y_{1:N}|\Theta^{\left(r\right)*}\right)}^{\frac{1}{T^{\left(r\right)}}}$ is obtained by iterative procedures of predictions, likelihood calculations and resampling; the latent variables $z_{n}$ of the current time step *n* are predicted and the likelihood is calculated for each particle, and resampling is performed according to the calculated likelihoods of particles at each time step. In the MH algorithm (Figure 2b), the sample candidate $\Theta^{\left(r\right)*}$ is determined to be accepted or rejected with the marginal likelihood $p{\left(y_{1:N}|\Theta^{\left(r\right)*}\right)}^{\frac{1}{T^{\left(r\right)}}}$. At this time, the target distribution becomes smooth as the temperature becomes high. As a result, it becomes easier to obtain samples from a wide range. Furthermore, exchanges between the samples at different temperatures are conducted in order to realize the transitions that are difficult depending on the initial values by the conventional PMMH method.

#### 2.3.2. Introducing the Replica Exchange Method into the PMMH Method

Here, we propose the REPMMH method to accurately estimate the distribution of parameters from observed data $y_{1:N}$. In our proposed method, we introduce replicas of parameters $\left\{\Theta \right\}=\left\{\Theta^{\left(1\right)},\Theta^{\left(2\right)},\ldots ,\Theta^{\left(r\right)},\ldots ,\Theta^{\left(R\right)}\right\}$ at different temperatures $T=\left[T^{\left(1\right)},T^{\left(2\right)},\ldots ,T^{\left(r\right)},\ldots ,T^{\left(R\right)}\right]$ and consider the extended joint marginal posterior distribution as follows:

(10) $$\pi_{\mathrm{EX}}\left(\left\{\Theta \right\}|y_{1:N}\right)=\prod_{r=1}^{R}\pi_{T^{\left(r\right)}}\left(\Theta^{\left(r\right)}|y_{1:N}\right),$$

where $\pi_{T^{\left(r\right)}}\left(\Theta^{\left(r\right)}|y_{1:N}\right)$ expresses the marginal posterior distribution at temperature $T^{\left(r\right)}$, which is expressed by using the original marginal posterior distribution $p\left(\Theta^{\left(r\right)}|y_{1:N}\right)$ of the parameter $\Theta^{\left(r\right)}$ at the temperature $T^{\left(1\right)}=1.0$ as follows:

(11) $$\pi_{T^{\left(r\right)}}\left(\Theta^{\left(r\right)}|y_{1:N}\right)=\frac{1}{z\left(T^{\left(r\right)}\right)}p{\left(\Theta^{\left(r\right)}|y_{1:N}\right)}^{\frac{1}{T^{\left(r\right)}}}\;\left(r=1,2,\ldots ,R\right),$$

where $z\left(T^{\left(r\right)}\right)$ is a partition function. Note that, as expressed in Equation (11), at sufficiently high temperatures, the distribution of parameters becomes closer to a uniform distribution, independent of the values of $y_{1:N}$. The distribution with $T^{\left(1\right)}=1.0$ corresponds to the original marginal posterior distribution $p\left(\Theta |y_{1:N}\right)$ to be investigated.

The marginal posterior distribution at each temperature $p\left(\Theta^{\left(r\right)}|y_{1:N}\right)$ is obtained using Bayes’ theorem as follows:

(12) $$p\left(\Theta^{\left(r\right)}|y_{1:N}\right)=\frac{p\left(y_{1:N}|\Theta^{\left(r\right)}\right)p\left(\Theta^{\left(r\right)}\right)}{p\left(y_{1:N}\right)}\;\left(r=1,2,\ldots ,R\right).$$

Namely, the marginal posterior distribution $p\left(\Theta^{\left(r\right)}|y_{1:N}\right)$ is proportional to a product of a marginal likelihood $p\left(y_{1:N}|\Theta^{\left(r\right)}\right)$ and a prior distribution $p\left(\Theta^{\left(r\right)}\right)$ of parameters $\Theta^{\left(r\right)}$. To obtain the marginal likelihood $p\left(y_{1:N}|\Theta^{\left(r\right)}\right)$, marginalization of the joint distribution at each temperature should be conducted as follows:

(13) $$p\left(y_{1:N}|\Theta^{\left(r\right)}\right)=\int p\left(y_{1:N},z_{1:N}^{\left(r\right)}|\Theta^{\left(r\right)}\right)dz_{1:N}^{\left(r\right)}\;\left(r=1,2,\ldots ,R\right),$$

where $z_{1:N}^{\left(r\right)}$ are the latent variables at the temperature $T^{\left(r\right)}$. By performing the SMC method for all the time steps at each temperature, the marginalization is conducted numerically.

As shown in Figure 2b,c, in the proposed method, the SMC method and the MH algorithm are conducted for each temperature. In the SMC method, the marginal likelihood of parameters $p\left(y_{1:N}|\Theta^{\left(r\right)*}\right)$ is determined by the numerical marginalization using the candidate of parameters $\Theta^{\left(r\right)*}$ proposed in the MH algorithms.

In the MH algorithm, the candidate of parameters $\Theta^{\left(r\right)*}$ is determined to be accepted or rejected at each temperature $T^{\left(r\right)}$ with the marginal posterior $\pi_{T^{\left(r\right)}}\left(\Theta^{\left(r\right)*}|y_{1:N}\right)$ (Figure 2b). Here, the acceptance probability $p_{\mathrm{accept}}^{\left(r\right)}$ at each temperature is calculated as follows:

(14) $$p_{\mathrm{accept}}^{\left(r\right)}=\min\left(1,\frac{p\left(y_{1:N}|\Theta^{\left(r\right)*}\right)p\left(\Theta^{\left(r\right)*}\right)}{p\left(y_{1:N}|\Theta^{\left(r\right)}\left[k-1\right]\right)p\left(\Theta^{\left(r\right)}\left[k-1\right]\right)}\frac{q\left(\Theta^{\left(r\right)}\left[k-1\right]|\Theta^{\left(r\right)*}\right)}{q\left(\Theta^{\left(r\right)*}|\Theta^{\left(r\right)}\left[k-1\right]\right)}\right).$$

Moreover, we exchange samples between different temperatures $T^{\left(r\right)}$ and $T^{\left(r+1\right)}$ according to the exchange probability as follows:

(15) $$p_{\mathrm{EX}}\left(\left\{\Theta \right\},{\left\{\Theta \right\}}^{*}\right)=\min\left(1,R_{\mathrm{EX}}\left(\left\{\Theta \right\},{\left\{\Theta \right\}}^{*}\right)\right),$$

(16) $$R_{\mathrm{EX}}\left(\left\{\Theta \right\},{\left\{\Theta \right\}}^{*}\right)=\frac{\pi_{\mathrm{EX}}\left({\left\{\Theta \right\}}^{*}|y_{1:N}\right)}{\pi_{\mathrm{EX}}\left(\left\{\Theta \right\}|y_{1:N}\right)},$$

where ${\left\{\Theta \right\}}^{*}$ is expressed as follows:

(17) $${\left\{\Theta \right\}}^{*}=\left\{\Theta^{\left(1\right)},\ldots ,\Theta^{\left(r+1\right)},\Theta^{\left(r\right)},\ldots ,\Theta^{\left(R\right)}\right\}.$$

Note that the exchange probability $p_{\mathrm{EX}}\left(\left\{\Theta \right\},{\left\{\Theta \right\}}^{*}\right)$ corresponds to the Metropolis criterion for proposing to exchange the samples between different temperatures $T^{\left(r\right)}$ and $T^{\left(r+1\right)}$. By deciding whether to accept or reject the proposed samples ${\left\{\Theta \right\}}^{*}$ with the Metropolis criterion of Equation (15), the transition probability $W\left(\left\{\Theta \right\}\to {\left\{\Theta \right\}}^{*}\right)$ for the exchange process satisfies the detailed balance condition as follows:

$$\begin{array}{ccc} & & \pi_{\mathrm{EX}}\left(\left\{\Theta \right\}|y_{1:N}\right)W\left(\left\{\Theta \right\}\to {\left\{\Theta \right\}}^{*}\right)\\ & & =\pi_{\mathrm{EX}}\left(\left\{\Theta \right\}|y_{1:N}\right)q\left({\left\{\Theta \right\}}^{*}|\left\{\Theta \right\}\right)p_{\mathrm{EX}}\left(\left\{\Theta \right\},{\left\{\Theta \right\}}^{*}\right)\\ & & =\min\left(\pi_{\mathrm{EX}}\left(\left\{\Theta \right\}|y_{1:N}\right)q\left({\left\{\Theta \right\}}^{*}|\left\{\Theta \right\}\right),\pi_{\mathrm{EX}}\left({\left\{\Theta \right\}}^{*}|y_{1:N}\right)q\left({\left\{\Theta \right\}}^{*}|\left\{\Theta \right\}\right)\right)\\ & & =\pi_{\mathrm{EX}}\left({\left\{\Theta \right\}}^{*}|y_{1:N}\right)q\left({\left\{\Theta \right\}}^{*}|\left\{\Theta \right\}\right)\min\left(\frac{\pi_{\mathrm{EX}}\left(\left\{\Theta \right\}|y_{1:N}\right)}{\pi_{\mathrm{EX}}\left({\left\{\Theta \right\}}^{*}|y_{1:N}\right)},1\right)\\ & & =\pi_{\mathrm{EX}}\left({\left\{\Theta \right\}}^{*}|y_{1:N}\right)q\left({\left\{\Theta \right\}}^{*}|\left\{\Theta \right\}\right)p_{\mathrm{EX}}\left({\left\{\Theta \right\}}^{*},\left\{\Theta \right\}\right)\\ & & =\pi_{\mathrm{EX}}\left({\left\{\Theta \right\}}^{*}|y_{1:N}\right)q\left(\left\{\Theta \right\}|{\left\{\Theta \right\}}^{*}\right)p_{\mathrm{EX}}\left({\left\{\Theta \right\}}^{*},\left\{\Theta \right\}\right)\\ & & =\pi_{\mathrm{EX}}\left({\left\{\Theta \right\}}^{*}|y_{1:N}\right)W\left({\left\{\Theta \right\}}^{*}\to \left\{\Theta \right\}\right), \end{array}$$

where $q\left({\left\{\Theta \right\}}^{*}|\left\{\Theta \right\}\right)$ is the proposed probability for ${\left\{\Theta \right\}}^{*}$ and the proposed probability of the exchange process is symmetric $q\left({\left\{\Theta \right\}}^{*}|\left\{\Theta \right\}\right)=q\left(\left\{\Theta \right\}|{\left\{\Theta \right\}}^{*}\right)$. Thus, since the exchange process in the proposed method satisfies the detailed balance condition, the proposed method can sample from the distribution $\pi_{\mathrm{EX}}\left(\left\{\Theta \right\}|y_{1:N}\right)$.

By this exchange process, the REPMMH method makes it possible to improve the problem of initial value dependence in the PMMH method. The sampled distributions of the replica $\pi_{T^{\left(r\right)}}\left(\Theta^{\left(r\right)}|y_{1:N}\right)$ at higher temperatures become closer to a uniform distribution ideally as follows:

(18) $$\lim_{T^{\left(r\right)}\to \infty }\pi_{T^{\left(r\right)}}\left(\Theta^{\left(r\right)}|y_{1:N}\right)=\lim_{T^{\left(r\right)}\to \infty }\frac{1}{z\left(T^{\left(r\right)}\right)}p{\left(\Theta^{\left(r\right)}|y_{1:N}\right)}^{\frac{1}{T^{\left(r\right)}}}\propto p{\left(\Theta^{\left(r\right)}|y_{1:N}\right)}^{0}=\mathrm{const}.$$

Therefore, in practice, it becomes possible to escape from local optima at sufficiently high temperatures (Figure 2b). Moreover, the samples may not stay in one local optimum since each replica is exchanged between the high temperature and low temperature repeatedly, and we can sample the parameters efficiently. We show the flow of our REPMMH method described above in Algorithm 2.

#### 2.3.3. Relations among Particle Markov Chain Monte Carlo Methods

We briefly summarize the differences among the conventional particle Markov chain Monte Carlo (PMCMC) methods and our proposed REPMMH method that can estimate parameters of a state space model in Table 1. The particle Gibbs (PG) method is another PMCMC method, and it samples latent variables and parameters in a state space model alternately by using Gibbs sampling [8,30]. The PMMH method combines the SMC method with the MH algorithm, whereas the PG method combines the SMC method with Gibbs sampling. While the SMC method is employed to calculate the marginal likelihood $p\left(y_{1:N}|\Theta \right)$ of parameters Θ in the PMMH method, the SMC method is employed to obtain samples of latent variables $z_{1:N}$ in the PG method [12]. The PMMH method directly targets the marginal posterior distribution $p\left(\Theta |y_{1:N}\right)$, whereas the PG method targets the joint posterior distribution $p\left(z_{1:N},\Theta |y_{1:N}\right)$ [12]. Note that the SMC method used in the PG method is called the conditional SMC method and uses the previous sample of latent variables $z_{1:N}\left[k-1\right]$ as a particle in the SMC method [12]. Furthermore, advanced versions of the PG method have been proposed, such as the particle Gibbs with ancestor sampling (PGAS) method for improving sampling efficiency and the replica exchange particle Gibbs with ancestor sampling (REPGAS) method to improve the initial value dependence [16,18,19,24]. Samples obtained by employing the PMMH method also have a problem of initial value dependence, similar to those obtained by employing the PG method, and it is considered that combining the PMMH method with the replica exchange method would be effective.
**Algorithm 2** Replica Exchange Particle Marginal Metropolis–Hastings (REPMMH) Method.1:initialize the parameters $\left\{\Theta \right\}\left[0\right]$2:**for**k=1,…,K**do**3:   **for** r=1,…,R **do**4:     draw the sample candidate of parameters $\Theta^{\left(r\right)*}∼q\left(\Theta^{\left(r\right)*}|\Theta^{\left(r\right)}\left[k-1\right]\right)$5:     calculate the marginal likelihood $p\left(y_{1:N}|\Theta^{\left(r\right)*}\right)$ by using the SMC method according to Equation (13)6:     calculate the acceptance probability $p_{\mathrm{accept}}^{\left(r\right)}$ with Equation (14)7:     draw a uniform random number $\alpha ∼U\left(0,1\right)$ ($U\left(a,b\right)$ is a uniform distribution with range [a,b))8:     **if** $\alpha \leq p_{\mathrm{accept}}^{\left(r\right)}$ **then**9:        set the sample of parameters $\Theta^{\left(r\right)}\left[k\right]\leftarrow \Theta^{\left(r\right)*}$10:     **else**11:        set the sample of parameters $\Theta^{\left(r\right)}\left[k\right]\leftarrow \Theta^{\left(r\right)}\left[k-1\right]$12:     **end if**13:   **end for**14:   choose the replica number $r_{\mathrm{EX}}\leftarrow 1$ or $r_{\mathrm{EX}}\leftarrow 2$ for the exchange15:   **repeat**16:     calculate exchange probability $p_{\mathrm{EX}}\left(\left\{\Theta \right\},{\left\{\Theta \right\}}^{*}\right)$ with Equation (15) for replica numbers $r_{\mathrm{EX}}$ and $r_{\mathrm{EX}}+1$17:     draw a uniform random number $\alpha_{\mathrm{EX}}∼U\left(0,1\right)$18:     **if** $\alpha_{\mathrm{EX}}\leq p_{\mathrm{EX}}\left(\left\{\Theta \right\},{\left\{\Theta \right\}}^{*}\right)$ **then**19:        exchange replicas $\left(\Theta^{\left(r_{\mathrm{EX}}\right)}\left[k\right],\Theta^{\left(r_{\mathrm{EX}}+1\right)}\left[k\right]\right)\leftarrow \left(\Theta^{\left(r_{\mathrm{EX}}+1\right)}\left[k\right],\Theta^{\left(r_{\mathrm{EX}}\right)}\left[k\right]\right)$20:     **end if**21:     set the replica number $r_{\mathrm{EX}}\leftarrow r_{\mathrm{EX}}+2$ for the exchange22:   **until** $r_{\mathrm{EX}}\leq R-1$23:**end for**24:**return**${\left\{\left\{\Theta \right\}\left[k\right]\right\}}_{k=1}^{K}$

## 3. Results

In this section, we show that by employing our proposed replica exchange particle marginal Metropolis–Hastings (REPMMH) method for the Izhikevich neuron model [41,42] and Lévy-driven stochastic volatility model [12,43,44], the marginal posterior distribution of parameters $p\left(\Theta |y_{1:N}\right)$ can be estimated from observations $y_{1:N}$, and we verify whether the REPMMH method can overcome the problem of initial value dependence in the particle marginal Metropolis–Hastings (PMMH) method. Moreover, we compare the sampling efficiency of the REPMMH method with that of the conventional methods, the PMMH method and the replica exchange particle Gibbs with ancestor sampling (REPGAS) method.

### 3.1. Izhikevich Neuron Model

To verify the effectiveness of our proposed method, we use the Izhikevich neuron model. The Izhikevich neuron model is a computational model for the membrane potential of a neuron [41,42]:

(19) $$\frac{dv}{dt}=0.04v^{2}+5v+140-u+I_{\mathrm{ext}}+\xi_{v}\left(t\right),$$

(20) $$\frac{du}{dt}=a\left(bv-u\right)+\xi_{u}\left(t\right),$$

where *v* is the membrane potential and *u* is the membrane recovery variable. $I_{\mathrm{ext}}$ is the input current from outside the neuron, and *a* and *b* are parameters in the Izhikevich neuron model that represent the characteristic of the neuron. In Equations (19) and (20), we consider additive white Gaussian noise terms $\xi_{v}\left(t\right)$ and $\xi_{u}\left(t\right)$ ($\langle \xi_{v}\left(t\right)\rangle =\langle \xi_{u}\left(t\right)\rangle =0$, $\langle \xi_{v}\left(t\right)\xi_{v}\left(s\right)\rangle =\sigma_{v}^{2}\delta \left(t-s\right)$, $\langle \xi_{u}\left(t\right)\xi_{u}\left(s\right)\rangle =\sigma_{u}^{2}\delta \left(t-s\right)$ and $\langle \xi_{v}\left(t\right)\xi_{u}\left(s\right)\rangle =0$, where δ(t) is the Dirac delta function). Here, standard deviations of the membrane potential and membrane recovery variable are expressed by $\sigma_{v}$ and $\sigma_{u}$, respectively. If the membrane potential *v* exceeds the threshold value $v_{\mathrm{th}}=30$, the membrane potential *v* and the membrane recovery variable *u* are reset to *c* and u+d, respectively, as follows:

$$\begin{array}{ccc} v & \leftarrow & c,\\ u & \leftarrow & u+d, \end{array}$$

where *c* and *d* are parameters representing the characteristic of the neuron.

**Figure 3:**
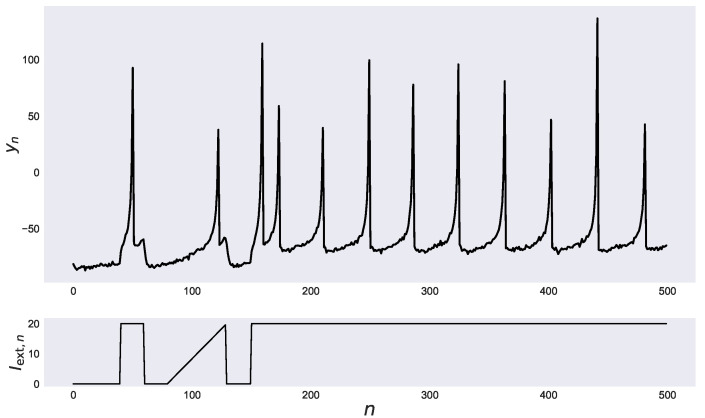
Observations and external inputs used to evaluate the proposed method. Observed membrane potential of the Izhikevich neuron model $y_{n}$ (**top**) in response to input current $I_{\mathrm{ext},n}$ (**bottom**) is shown.

Here, we assume that the observations $y_{1:N}$ are the membrane potentials with Gaussian observation noise, and we estimate the parameters $\Theta =\left\{a,b,c,d\right\}$ from only the observations $y_{1:N}$. We use the true parameters $\Theta =\left\{a,b,c,d\right\}=\left\{0.02,0.2,-65,6\right\}$ and the number of data $N=5.0\times 10^{2}$ to generate data. In the system model, the means and the variances of the Gaussian noise are $\left\{\mu_{v},\sigma_{v}^{2}\right\}=\left\{0,0.25\right\}$ and $\left\{\mu_{u},\sigma_{u}^{2}\right\}=\left\{0,10^{-4}\right\}$. In the observation model, the mean and the variance of the Gaussian noise are $\left\{\mu_{y},\sigma_{y}^{2}\right\}=\left\{0,1\right\}$. We show the generated data from the Izhikevich neuron model in Figure 3. In Figure 3, complex spike activities with different inter-spike intervals and different peaks are seen in response to external inputs. We assume that only one-dimensional time-series of observed data $y_{n}$ and external inputs can be used, while the latent dynamics are governed by two-dimensional nonlinear dynamical systems with four parameters $\Theta =\left\{a,b,c,d\right\}$. We employ our REPMMH method and the conventional methods, the PMMH method and the REPGAS method, for the generated data in Figure 3 to estimate the posterior distribution of the parameters $\Theta =\left\{a,b,c,d\right\}$. In all methods, the initial values of the parameters $\Theta \left[0\right]$ are $\left\{a,b,c,d\right\}=\left\{0.025,0.15,-60,5.5\right\}$, the number of samples *K* is $10^{6}$, the number of burn-in samples $K_{\mathrm{burn}-\mathrm{in}}$ is $10^{6}$, and the number of particles *M* is 50. In our REPMMH method and the REPGAS method, the number of temperatures *R* is 64.

**Figure 4:**
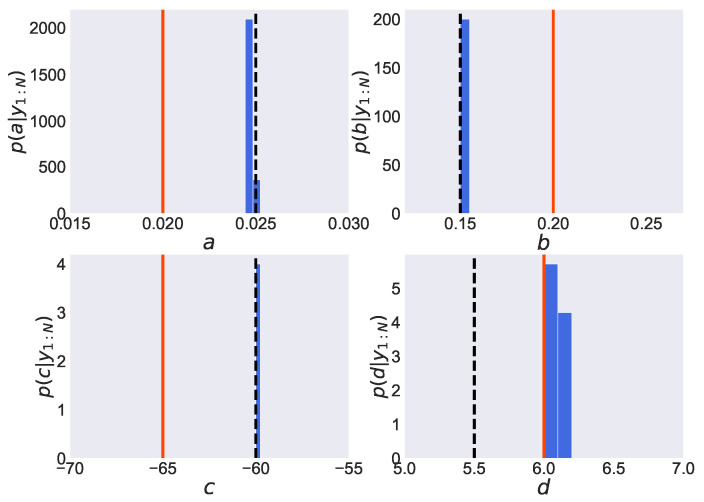
Estimated posterior distributions obtained by employing the PMMH method in the Izhikevich neuron model. In each graph, the estimated probability density function of the parameter (*a*, *b*, *c* and *d*) is shown by the blue histogram. The red solid and black dashed lines express the true and initial values, respectively.

We show in Figure 4 the estimated posterior distribution of parameters $p\left(\Theta |y_{1:N}\right)$ obtained by employing the PMMH method. In each graph, the vertical axis expresses the value of the probability density function, while the horizontal axis expresses the values of parameters *a*, *b*, *c* and *d*. Furthermore, the solid lines represent the true values, the dashed lines represent the initial values, and the histograms represent the estimated posterior distributions of the parameters. From Figure 4, we find that a peak of the estimated posterior distribution of parameter *d*, $p\left(d|y_{1:N}\right)$, is located around its true value d=6.0. However, the maximum values of the estimated posterior distribution of the other three parameters, *a*, *b* and *c*, remain around their initial values (a=0.025, b=0.15, c=−60), which are far from their true values (a=0.020, b=0.20, c=−65). Thus, the joint posterior distribution of four parameters is found to be not adequately estimated. From this result, the samples in the PMMH method are considered to remain in the local optimum since the initial values are far from the true value.

**Figure 5:**
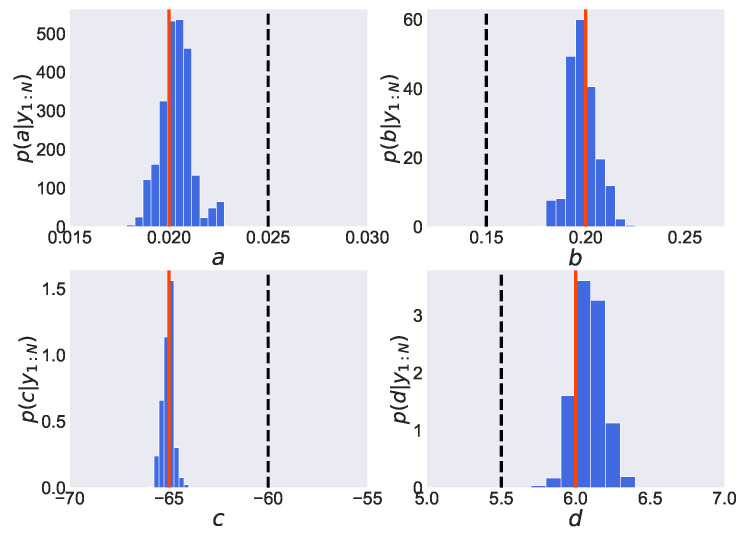
Estimated posterior distributions obtained by employing the REPMMH method in the Izhikevich neuron model. See also the captions of the figure and subfigures for Figure 4.

We show in Figure 5 the estimated posterior distribution of parameters $p\left(\Theta |y_{1:N}\right)$ obtained by employing the REPMMH method. From Figure 5, we find that the maximum values of the estimated posterior distribution of parameters are located around the true values (a=0.020, b=0.20, c=−65, d=6.0), even though the initial values of the parameters (a=0.025, b=0.15, c=−60, d=5.5) are set to be far from the true values. This improvement in estimation accuracy would be induced by combination with the replica exchange method. It is easier to obtain samples from a wider range since the replica exchange method allows samples to pass through high temperatures. From these results, we find that the problem of initial value dependence in the PMMH method is improved by employing our proposed method.

**Figure 6:**
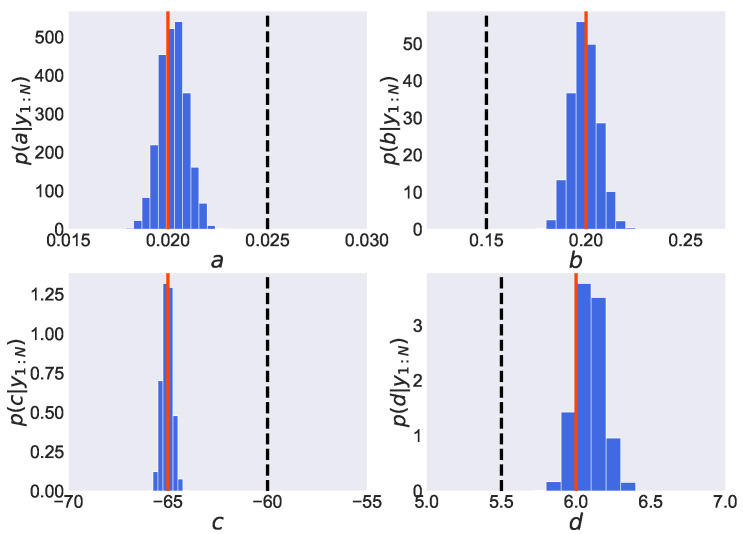
Estimated posterior distributions obtained by employing the replica exchange particle Gibbs with ancestor sampling (REPGAS) method in the Izhikevich neuron model. See also the captions of the figure and subfigures for Figure 4.

Moreover, we show in Figure 6 the estimated posterior distribution of parameters $p\left(\Theta |y_{1:N}\right)$ obtained by employing the REPGAS method. As shown in this figure, the distributions are estimated to be almost the same as those obtained by employing the REPMMH method, which indicates that the true values are estimated properly.

**Figure 7:**
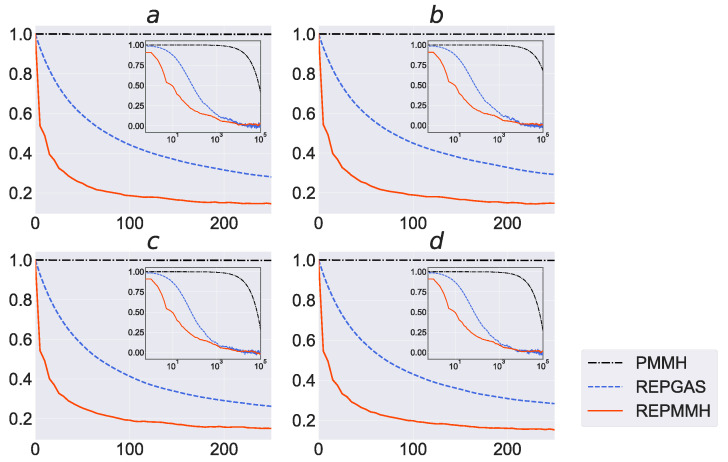
Autocorrelation as a function of the lag length for parameters *a*, *b*, *c* and *d* in the Izhikevich neuron model. Results for the PMMH method (black dashed-dotted line), the REPGAS method (blue dashed line) and the REPMMH method (red solid line) are shown. Each inset figure represents the result when the horizontal axis is the logarithmic scale. In results obtained by the REPGAS method and the REPMMH method, samples at $T^{\left(1\right)}=1.0$ were used.

In order to investigate the efficiency of sampling parameters in the proposed method and existing methods, we show in Figure 7 the autocorrelation function results calculated using the samples of the PMMH method, the REPGAS method and our proposed REPMMH method. In all the parameters *a*, *b*, *c* and *d*, the decay of the autocorrelation in the REPMMH samples is faster using the REPMMH samples than those calculated using the PMMH samples and the REPGAS samples. The time constant of the autocorrelation function has a strong influence on the convergence time of the PMCMC method. The time constants of the autocorrelation functions for the REPMMH samples are around 20 for all parameters *a*, *b*, *c* and *d*, while those of the autocorrelation functions for the PMMH samples are more than $10^{5}$, as shown in Figure 7. Since the computational cost of the exchange process in the REPMMH method is very small compared to the computational cost of the SMC method, the computational cost of the REPMMH method is approximately R=64 times the computational cost of the PMMH method. Nevertheless, the REPMMH method drastically improves the sampling efficiency compared to the increase in the computational cost; the REPMMH method is R=64 times more computationally expensive than the PMMH method, while the effective sample size of the REPMMH method is much larger (around $10^{3}$ times larger) than that of the PMMH method.

When the same number of temperatures and particles is used, the REPGAS method is more computationally expensive than the REPMMH method since the REPGAS method requires the sampling of the latent variables $z_{1:N}$ and the ancestor sampling, which considers sampling of the latent variables $z_{1:N}$ not only in the forward direction but also in the backward direction in the conditional SMC method. Nevertheless, the REPMMH method has high sampling efficiency compared to the REPGAS method. Thus, we find that the sampling efficiency of our proposed REPMMH method is higher than that of the conventional methods.

Moreover, in order to evaluate the influence of the number of temperatures *R* and the number of particles *M* on the estimated results, we compare the estimated results in various settings. We show the estimated results with the numbers of temperatures R=1, 4, 16 and 64 in Table 2. Table 2 shows the mode values of the estimated distributions, the standard deviations (Std) of the estimated distributions and the values of autocorrelation functions (ACF) with the lag length 30 for the numbers of temperatures R=1, 4, 16 and 64. Note that the numbers of particles *M* are 50 in all cases and the maximum value of temperature is fixed at $T^{\left(R\right)}=1.1^{63}$ for R>1.

As mentioned above, for the number of temperatures R=1, we can estimate the parameter *d* around the true value d=6.0, while the other three parameters *a*, *b* and *c* remain at their initial values (a=0.025, b=0.15, c=−60). We also find that the samples of parameters *a*, *b* and *c* cannot move enough from their initial values since the values of the standard deviations are very small. For the number of temperatures R=4, we find the samples can escape the local optima and we can estimate the true values of all parameters *a*, *b*, *c* and *d* accurately due to the high temperatures that allow escape from the local optima. However, since the values of the autocorrelation functions are close to 1.0, we need a large number of samples in order to estimate the shape of the distribution $p\left(\Theta |y_{1:N}\right)$. On the other hand, we find that the values of the autocorrelation functions are smaller for R=16 and 64.

We show the estimated results with the numbers of particles M=10, 20, 30, 40 and 50 in Table 3. Note that the numbers of temperatures *R* are 64 in all cases. For the numbers of particles M=10 and 20, the estimated values of the parameters *a*, *b*, *c* and *d* are far from the true values (a=0.020, b=0.20, c=−65, d=6.0). We consider that these results are due to the low approximation accuracy of the marginal likelihood $p\left(y_{1:N}|\Theta \right)$ in the SMC method with too small numbers of particles. For the numbers of particles M≥30, we can estimate the true values of parameters *a*, *b*, *c* and *d*. Since there is no significant difference between the mode values, the standard deviations and the values of the autocorrelation functions for the numbers of particles M=30, 40 and 50, we consider that the number of particles *M* is sufficient for this problem if it is above 30.

### 3.2. Lévy-Driven Stochastic Volatility Model

Next, we also verify the effectiveness of the proposed method using the Lévy-driven stochastic volatility model [12,43,44]. In this model, the dynamics of the logarithm of asset price $y^{*}\left(t\right)$ are represented by the following differential equation:

(21) $$dy^{*}\left(t\right)=\left\{\mu +\beta \sigma^{2}\left(t\right)\right\}dt+\sigma \left(t\right)dB\left(t\right),$$

where μ is the drift parameter and β is the risk premium. $B\left(t\right)$ is the Brownian motion and $\sigma^{2}\left(t\right)$ represents the volatility. The dynamics of the volatility $\sigma^{2}\left(t\right)$ are modeled by the following Lévy-driven Ornstein–Unlenbeck process:

(22) $$d\sigma^{2}\left(t\right)=-\lambda \sigma^{2}\left(t\right)dt+dz\left(\lambda t\right),$$

where λ is a positive constant and $z\left(t\right)$ is a non-Gaussian Lévy process with positive increments. The observation at the time step *n*, $y_{n}$, in this model is obtained by the following Gaussian distribution:

(23) $$y_{n}∼N\left(\mu \Delta +\beta \sigma_{n}^{2},\sigma_{n}^{2}\right),$$

where Δ is the length of the time interval.

The stochastic volatility models are numerically investigated by using discretized dynamical models [12,43,44], and the estimation algorithm for the parameters of the stochastic volatility models has been investigated using such the discretized ones [12]. The integrated volatility $\sigma_{n}^{2}$ at the time step *n* is calculated as follows:

(24) $$\begin{array}{ccc} \sigma_{n}^{2} & = & \int_{\left(n-1\right)\Delta }^{n\Delta }\sigma^{2}\left(u\right)du\\ & = & \lambda^{-1}\left[z\left(\lambda n\Delta \right)-\sigma^{2}\left(n\Delta \right)-z\left\{\lambda \left(n-1\right)\Delta \right\}+\sigma^{2}\left\{\left(n-1\right)\Delta \right\}\right], \end{array}$$

where $\sigma^{2}\left(n\Delta \right)$ and $z\left(\lambda n\Delta \right)$ are, respectively, represented as follows:

(25) $$\sigma^{2}\left(n\Delta \right)=\exp\left(-\lambda \Delta \right)\sigma^{2}\left\{\left(n-1\right)\Delta \right\}+\eta_{\sigma ,n},$$

(26) $$z\left(\lambda n\Delta \right)=z\left\{\lambda \left(n-1\right)\Delta \right\}+\eta_{z,n}.$$

Here, we address the case where the volatility $\sigma^{2}\left(t\right)$ follows a tempered stable marginal distribution [44]. Following [2,44], $\eta_{\sigma ,n}$ and $\eta_{z,n}$ are obtained as follows:

(27) $$\eta_{\sigma ,n}=\sum_{i=1}^{\infty }\min\left({\left(\frac{a_{i}\kappa }{A\lambda \Delta }\right)}^{-1/\kappa },e_{i}v_{i}^{1/\kappa }\right)\exp\left(-\lambda \Delta r_{i}\right)+\sum_{i=1}^{N\left(\lambda \Delta \right)}c_{i}\exp\left(-\lambda \Delta r_{i}^{*}\right),$$

(28) $$\eta_{z,n}=\sum_{i=1}^{\infty }\min\left({\left(\frac{a_{i}\kappa }{A\lambda \Delta }\right)}^{-1/\kappa },e_{i}v_{i}^{1/\kappa }\right)+\sum_{i=1}^{N\left(\lambda \Delta \right)}c_{i},$$

where $A=2^{\kappa }\delta \kappa^{2}/\Gamma \left(1-\kappa \right)$, $a_{1}<a_{2}<\ldots$ are the arrival times of a Poisson process with intensity 1, $e_{1},e_{2},\ldots$ are independent and identically distributed exponential random variables with mean $2\gamma^{-1/\kappa }$, and $v_{1},v_{2},\ldots$, $r_{1},r_{2},\ldots$ and $r_{1}^{*},r_{2}^{*},\ldots$ are standard uniform random variables. $c_{1},c_{2},\ldots$ are obtained from a gamma distribution with the shape parameter 1−κ and the scale parameter $2\gamma^{-1/\kappa }$, and $N\left(\lambda \Delta \right)$ is obtained from a Poisson distribution with mean λΔδγκ. Here, κ, δ, γ and λ are the parameters $\Theta =\left\{\kappa ,\delta ,\gamma ,\lambda \right\}$ to be estimated.

In this paper, we employ the proposed method and the PMMH method for the stochastic volatility model. The PMMH-based methods, including the proposed method, can be applied to complex models such as the Lévy-driven stochastic volatility model, as long as the probability density of the observation model can be calculated. On the other hand, the PG-based method is difficult to apply to the stochastic volatility model due to the need to calculate the probability density of the system model [12]. Following [12], we use the true parameters $\Theta =\left\{\kappa ,\delta ,\gamma ,\lambda \right\}=\left\{0.5,1.41,2.83,0.1\right\}$, the number of data $N=4.0\times 10^{2}$ and the time interval of length Δ=1.0 to generate data. In order to estimate the parameters Θ, we use the initial values of the parameters $\Theta \left[0\right]=\left\{0.25,7.41,9.83,1.5\right\}$, the number of samples $K=1.5\times 10^{5}$, the number of burn-in samples $K_{\mathrm{burn}-\mathrm{in}}=10^{5}$ and the number of particles M=200 in both the proposed REPMMH method and the PMMH method. In the REPMMH method, the number of temperatures *R* is 64.

**Figure 8:**
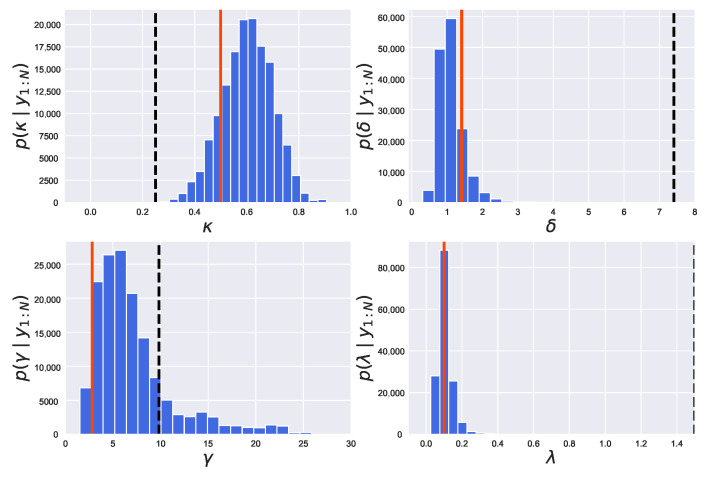
Estimated posterior distributions obtained by employing the PMMH method in the Lévy-driven stochastic volatility model. In each graph, the estimated probability density function of parameters (κ, δ, γ and λ) is shown by the blue histogram. The red solid and black dashed lines express the true and initial values, respectively.

We show in Figure 8 the estimated posterior distribution of parameters $p\left(\Theta |y_{1:N}\right)$ obtained by employing the PMMH method. From Figure 8, we find that a peak of the estimated posterior distribution of parameter λ, $p\left(\lambda |y_{1:N}\right)$, is located around its true value λ=0.1. However, the maximum values of the estimated posterior distribution of the other three parameters, κ, δ and γ, are far from their true values (κ=0.5, δ=1.41, γ=2.83). Thus, the joint posterior distribution of the four parameters is found to be not adequately estimated. It is considered that the target distribution is not reached with a small number of samples since the sampling efficiency of the PMMH method is low.

**Figure 9:**
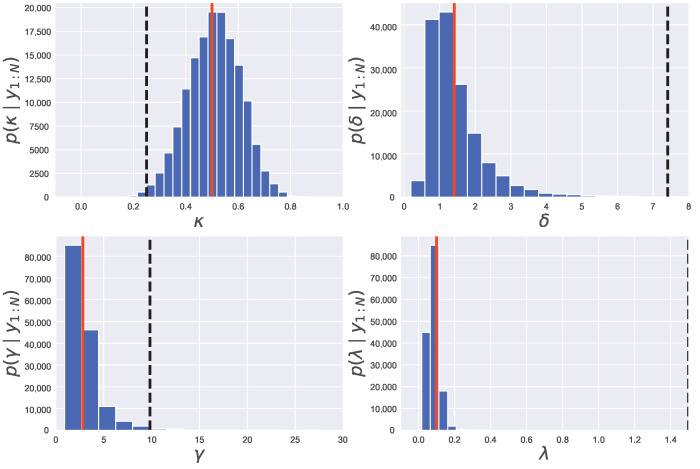
Estimated posterior distributions obtained by employing the REPMMH method in the Lévy-driven stochastic volatility model. See also the captions of the figure and subfigures for Figure 8.

We show in Figure 9 the estimated posterior distributions of parameters $p\left(\Theta |y_{1:N}\right)$ obtained by employing the REPMMH method. From Figure 9, we find that the true values of parameters $\Theta =\left\{\kappa ,\delta ,\gamma ,\lambda \right\}=\left\{0.5,1.41,2.83,0.1\right\}$ are estimated appropriately by using the same number of samples and the same initial values $\Theta \left[0\right]=\left\{0.25,7.41,9.83,1.5\right\}$ as the PMMH method. The results in Figure 8 and Figure 9 show that our REPMMH method has higher sampling efficiency than the PMMH method.

**Figure 10:**
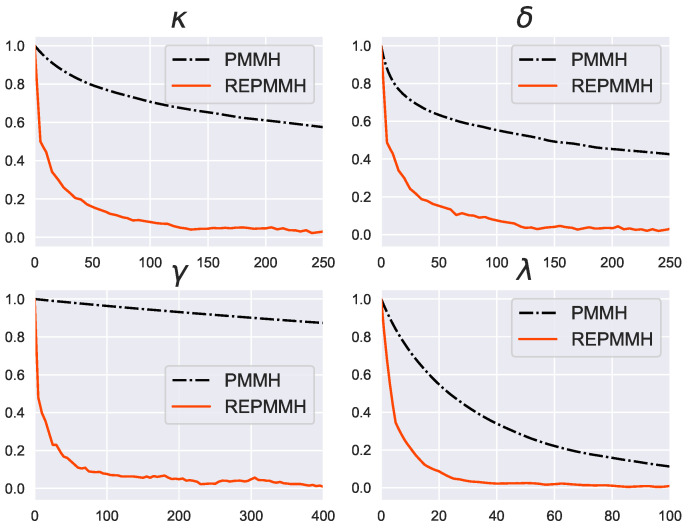
Autocorrelation as a function of the lag length for parameters κ, δ, γ and λ in the Lévy-driven stochastic volatility model. Results for the PMMH method (black dashed-dotted line) and the REPMMH method at $T^{\left(1\right)}=1.0$ (red solid line) are shown.

Moreover, we show in Figure 10 the autocorrelation function results calculated using the samples of the PMMH method and the REPMMH method. In all parameters κ, δ, γ and λ, the decay of the autocorrelation is faster using the REPMMH samples than that using the PMMH samples. As shown in Figure 10, the time constants of the autocorrelation functions for the REPMMH samples are less than 15 for all parameters κ, δ, γ and λ, while the time constant of the autocorrelation functions for the PMMH samples for the parameter γ is more than $3.0\times 10^{3}$. As mentioned above, since the computational cost of the REPMMH method is approximately R=64 times the computational cost of the PMMH method, the REPMMH method improves the sampling efficiency compared to the increase in the computational cost.

## 4. Concluding Remarks

In this paper, we have proposed the replica exchange particle marginal Metropolis–Hastings (REPMMH) method in order to estimate the marginal posterior distribution of parameters $p\left(\Theta |y_{1:N}\right)$ of the state space model. Our proposed method can be applied to complex models such as the Lévy-driven stochastic volatility model, even if the probability densities of the system models cannot be calculated explicitly. By the proposed method, we introduce the exchange between samples of model parameters Θ at different temperatures and realize an efficient sampling method for model parameters governing the nonlinear dynamical systems.

Using nonlinear dynamical models such as the Izhikevich neuron model and Lévy-driven stochastic volatility model, we show that our proposed REPMMH method can improve the problem of initial value dependence of the particle marginal Metropolis–Hastings (PMMH) method. The results have shown that the proposed REPMMH method accurately estimates the marginal posterior distribution of parameters. Moreover, by comparing the autocorrelation functions of the obtained samples, it has been also shown that our proposed REPMMH method can sample more efficiently than the conventional methods. In the replica exchange particle Gibbs with ancestor sampling (REPGAS) method, the next sample of latent variables is obtained under the strong influence of the current sample of latent variables. On the other hand, in the REPMMH method, the correlation of the latent variables between the current and next steps is low since the REPMMH method only calculates the marginal likelihood of the next step, regardless of the latent variables obtained in the current step. Therefore, it is considered that the REPMMH method can sample parameters more efficiently than the REPGAS method.

In this paper, although we conducted the experiments by using two specific dynamical models: the Izhikevich neuron model and the Lévy-driven stochastic volatility model, the proposed REPMMH method can be applied to various dynamical systems described by ordinary or partial differential equations. Although the proposed method can be applied to any ordinary or partial differential equations that can be represented as state space models, applications of the proposed method are difficult when the system models for the dynamical systems or the observation models are completely unknown. In such cases, we consider that combining the proposed method with non-parametric Bayesian methods is effective. We leave this for future work.

## Figures and Tables

**Table 1:** The PMCMC methods for estimating parameters in a state space model.

Method	Target Distribution	Overview
PG	$p\left(z_{1:N},\Theta |y_{1:N}\right)$	Sample parameters Θ and latent variables $z_{1:N}$ alternately with Gibbs sampling for targeting the joint posterior distribution $p\left(z_{1:N},\Theta |y_{1:N}\right)$ Note that the SMC method is used for sampling latent variables $z_{1:N}$. The SMC method used in the PG method is called the conditional SMC method and uses the previous sample of latent variables $z_{1:N}\left[k-1\right]$ as a particle in the SMC method [12].
PGAS	$p\left(z_{1:N},\Theta |y_{1:N}\right)$	Sample latent variables $z_{1:N}$ not only in the forward direction but also in the backward direction in the PG method [16,18,19].
REPGAS	$p\left(z_{1:N},\Theta |y_{1:N}\right)$	Improve the problem of initial value dependence in the PGAS method by combining the replica exchange method and the PGAS method [24].
PMMH	$p\left(\Theta |y_{1:N}\right)$	Sample parameters Θ with the MH algorithm for targeting directly the marginal posterior distribution $p\left(\Theta |y_{1:N}\right)$ obtainedby marginalization over the distribution of latent variables $z_{1:N}$. Note that the SMC method is used to calculate the marginal likelihood $p\left(y_{1:N}|\Theta \right)$ [12].
REPMMH	$p\left(\Theta |y_{1:N}\right)$	Improve the problem of initial value dependence in the PMMH method by combining the replica exchange method and the PMMH method.

**Table 2:** The estimated results with the numbers of temperatures R=1, 4, 16 and 64.

Parameter		R=1	R=4	R=16	R=64
a=0.020	Mode	0.0251	0.0200	0.0205	0.0205
	Std	$5.5\times 10^{-5}$	$6.9\times 10^{-4}$	$6.7\times 10^{-4}$	$7.8\times 10^{-4}$
	ACF	0.9999	0.9914	0.5175	0.3074
b=0.20	Mode	0.155	0.200	0.200	0.200
	Std	$3.0\times 10^{-4}$	$7.2\times 10^{-3}$	$7.3\times 10^{-3}$	$7.0\times 10^{-3}$
	ACF	0.9999	0.9919	0.5773	0.3082
c=−65	Mode	−60.0	−64.75	−65.0	−65.0
	Std	$2.1\times 10^{-3}$	$2.8\times 10^{-1}$	$2.5\times 10^{-1}$	$2.6\times 10^{-1}$
	ACF	0.9999	0.9926	0.5359	0.3117
d=6.0	Mode	6.10	6.10	6.05	6.05
	Std	$2.0\times 10^{-2}$	$8.9\times 10^{-2}$	$9.8\times 10^{-2}$	$9.8\times 10^{-2}$
	ACF	0.9999	0.9928	0.5222	0.3176

**Table 3:** The estimated results with the numbers of particles M=10, 20, 30, 40 and 50.

Parameter		M=10	M=20	M=30	M=40	M=50
a=0.020	Mode	0.0220	0.0210	0.0200	0.0200	0.0205
	Std	$5.9\times 10^{-4}$	$7.5\times 10^{-4}$	$7.3\times 10^{-4}$	$6.7\times 10^{-4}$	$7.8\times 10^{-4}$
	ACF	0.5548	0.2707	0.3072	0.3391	0.3074
b=0.20	Mode	0.195	0.190	0.200	0.195	0.200
	Std	$4.9\times 10^{-3}$	$8.3\times 10^{-3}$	$7.1\times 10^{-3}$	$5.9\times 10^{-3}$	$7.0\times 10^{-3}$
	ACF	0.2738	0.2611	0.2932	0.3352	0.3082
c=−65	Mode	−60.75	−64.50	−65.00	−65.00	−65.00
	Std	$2.9\times 10^{-1}$	$3.7\times 10^{-1}$	$2.7\times 10^{-1}$	$2.8\times 10^{-1}$	$2.6\times 10^{-1}$
	ACF	0.9223	0.3071	0.2793	0.3701	0.3117
d=6.0	Mode	6.50	6.10	6.10	6.05	6.05
	Std	$1.4\times 10^{-1}$	$8.2\times 10^{-2}$	$1.0\times 10^{-1}$	$9.3\times 10^{-2}$	$9.8\times 10^{-2}$
	ACF	0.6096	0.2750	0.3220	0.2985	0.3176

## Data Availability

Not applicable.

## Abbreviations

The following abbreviations are used in this manuscript:

PMMH	Particle Marginal Metropolis–Hastings
REPMMH	Replica Exchange Particle Marginal Metropolis–Hastings
SMC	Sequential Monte Carlo
EM	Expectation–Maximization
MCMC	Markov Chain Monte Carlo
PMCMC	Particle Markov Chain Monte Carlo
MH	Metropolis–Hastings
PG	Particle Gibbs
PGAS	Particle Gibbs with Ancestor Sampling
REPGAS	Replica Exchange Particle Gibbs with Ancestor Sampling
